# The isotopic niche of Atlantic, biting marine mammals and its relationship to skull morphology and body size

**DOI:** 10.1038/s41598-021-94610-w

**Published:** 2021-07-26

**Authors:** Massimiliano Drago, Marco Signaroli, Meica Valdivia, Enrique M. González, Asunción Borrell, Alex Aguilar, Luis Cardona

**Affiliations:** 1 Department of Evolutionary Biology, Ecology and Environmental Sciences, Biodiversity Research Institute (IRBio), University of Barcelona, Av. Diagonal 643, 08028 Barcelona, Spain; 2 National Museum of Natural History (MNHN), 25 de Mayo 582, 11000 Montevideo, Uruguay

**Keywords:** Ecology, Stable isotope analysis, Tropical ecology

## Abstract

Understanding the trophic niches of marine apex predators is necessary to understand interactions between species and to achieve sustainable, ecosystem-based fisheries management. Here, we review the stable carbon and nitrogen isotope ratios for biting marine mammals inhabiting the Atlantic Ocean to test the hypothesis that the relative position of each species within the isospace is rather invariant and that common and predictable patterns of resource partitioning exists because of constrains imposed by body size and skull morphology. Furthermore, we analyze in detail two species-rich communities to test the hypotheses that marine mammals are gape limited and that trophic position increases with gape size. The isotopic niches of species were highly consistent across regions and the topology of the community within the isospace was well conserved across the Atlantic Ocean. Furthermore, pinnipeds exhibited a much lower diversity of isotopic niches than odontocetes. Results also revealed body size as a poor predictor of the isotopic niche, a modest role of skull morphology in determining it, no evidence of gape limitation and little overlap in the isotopic niche of sympatric species. The overall evidence suggests limited trophic flexibility for most species and low ecological redundancy, which should be considered for ecosystem-based fisheries management.

## Introduction

Pinnipeds and odontocetes are apex predators in marine food webs worldwide, relying largely on fishes and cephalopods^1,2^. As a result, they often interact with fisheries^3^, and precise understanding of their trophic niches is necessary to model the consequences of such interactions, minimize conflicts and achieve sustainable, ecosystem based fisheries management^4–8^.

Traditional methods to study the diet of marine mammals have been stomach content analysis and scat analysis (the last only used routinely for pinnipeds). These approaches have a very high taxonomic resolution and often reveal a broad diversity of prey through time and space in the diet of the same species in response to changes in the make-up of the local fish and squid communities^e.g.^ ^9–11^. Such variability may suggest that most pinnipeds and odontocetes are opportunistic predators with broad fundamental ecological niches and variable positions in food webs. However, morphological analysis suggests that food acquisition in both groups is strongly determined by body size and skull morphology because of restrictions imposed on the aerobic dive limit and the feeding mode^12–19^.

Most pinnipeds and many odontocetes are biting feeders, as they seize or grasp their prey with the teeth^12–17,20^. All extant mysticetes and a few pinnipeds are filter feeders, and deep diving odontocetes and elephant seals pursuing mesopelagic squids, as well as a few pinnipeds foraging on benthic molluscs, are suction feeders^12,13,16–20^. Biting feeding is associated to a much smaller body mass, with only two species being larger than 1.000 kg, namely killer whales *Orcinus orca* and false killer whales *Pseudorca crassidens*^14,15^. This results into a non-monotonic relationship between trophic position and body size in marine animal communities that include marine mammal representatives of these three feeding modes because the trophic position of the massive mysticetes is lower than that of biting marine mammals^21^. Furthermore, skull shape has been suggested to be more important than body size in determining prey size in biting odontocetes, with longirostrine species consuming comparatively smaller prey and brevirostrine species with short, wide and tall skull consuming comparatively medium to larger prey^14,15^. This is because the existence of a trade-off between hydrodynamic performance and bite force production, which are maximized by longirostrine and brevirostrine skull morphologies respectively^14,15^. Furthermore, biting marine mammals mostly capture prey that can be swallowed whole^17,22,23^, and hence are thought to be gape limited^21^. Nevertheless, longirostrine odontocetes show preference for feeding on prey well below their maximum prey size because of hydrodynamic constrains^14^ and there is increasing evidence that many pinnipeds and several odontocetes may tear apart their prey^16,24^ thus overcoming the limits on prey size imposed by their gape breadth.

It follows from the above that the apparent dietary flexibility of marine mammals suggested by the broad taxonomic diversity of their prey, as revealed by stomach contents and scat analysis, could be constrained by morphology within much narrower ecological limits than usually believed. If so, the fundamental trophic niches of marine mammals would be rather narrow and hence their trophic positions within food webs would be rather invariant across time and space. It should be noted, however, that skull morphology imposes hydrodynamic constrains that may decouple mouth gape from prey size^14,15^. This is because brevirostrine skulls are less efficient in the capture of fast swimming prey than longirostrine skulls, which on the contrary as associated to rather weak mandibles^14,15^.

Stable isotope analysis offers a convenient approach to address these issues. Study of diet through stable isotope analysis lacks the taxonomic resolution of stomach content and scat analyses, but stable isotope ratios in animal tissues integrate dietary information through variable time spans, depending on the tissue turnover rate^25^. This alleviates the extremely short time resolution of stomach and scat contents, which often reveal the composition of just the last few meals. The stable isotopes of carbon (C) and nitrogen (N) are particularly convenient because they allow the calculation of simple metrics that encapsulate key information about the ecological niche of predators^26–28^. Thus, the C stable isotope ratio is informative about the primary source of carbon and decreases consistently along an onshore-offshore gradient in aquatic ecosystems^29^, and the N stable isotope ratio increases consistently along the food web providing a convenient and simple method to assess the trophic position of species^30^.

Here, we review the stable isotope ratios of C and N for biting pinnipeds and odontocetes inhabiting the Atlantic Ocean, from cold temperate Europe to subantarctic South America, to test the hypotheses that (i) the relative position of each species within the isospace is rather invariant and (ii) that common and predictable patterns of resource partitioning exist across communities because of restrictions associated to body size and skull morphology. Furthermore, we analyze in detail two species-rich communities from Mauritania and Uruguay to assess the actual degree of overlap in the isotopic niches of species and test the hypothesis that marine mammals are gape limited and, hence, that trophic position increases with gape size.

## Methods

### Literature search

**Figure 1:**
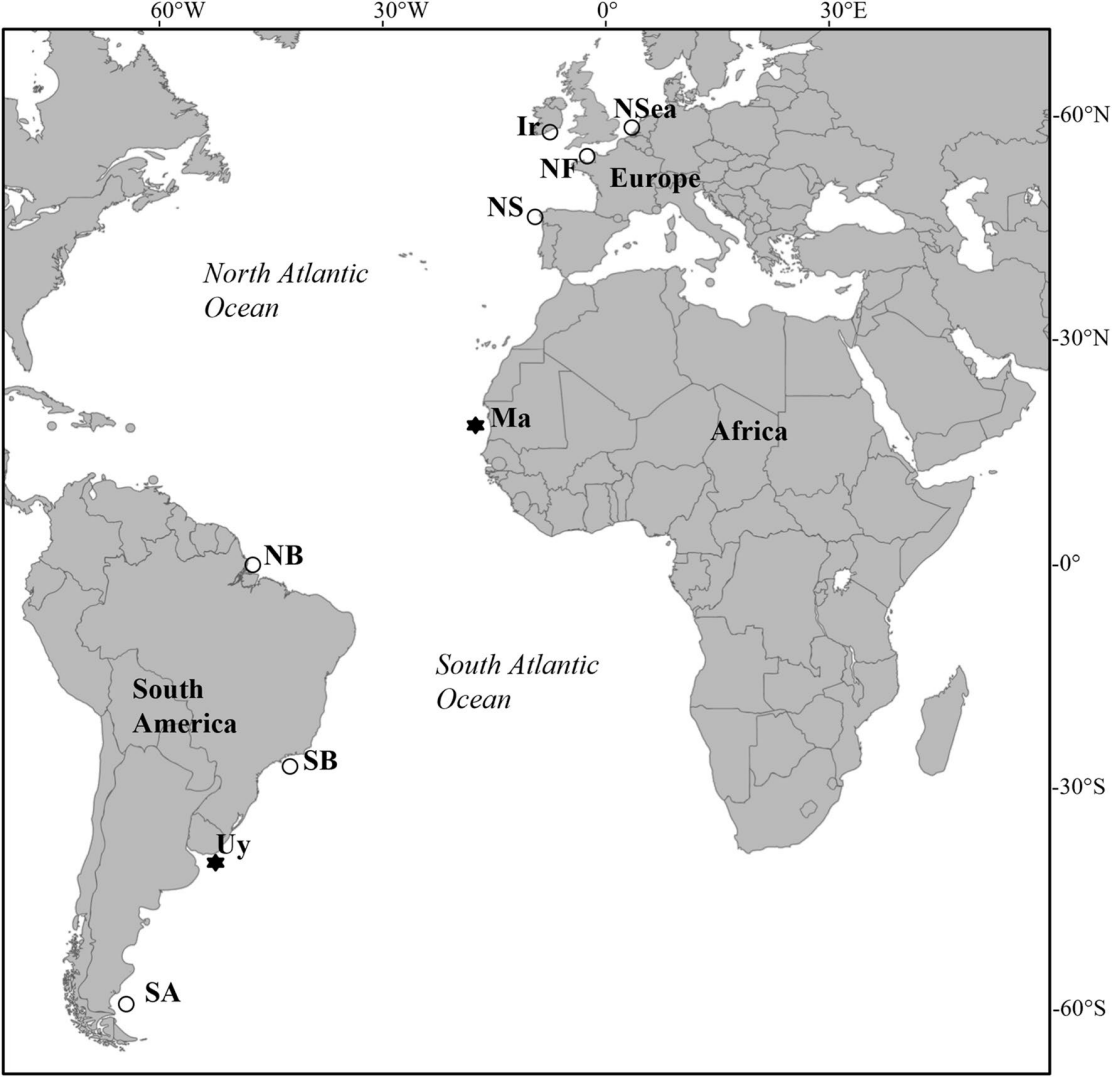
Map showing the geographic positions of the studies containing stable isotope information of marine mammal species used in this study. Ireland (Ir), Northern France (NF), North Sea (NSea), Northern Spain (NS), Mauritania (Ma), Northern Brazil (NB), Southern Brazil (SB), Uruguay (Uy) and Southern Argentina (SA). In asterisk, the two compared marine mammal communities.

Stable isotope ratios of C and N have been compiled from several published studies addressing resource partitioning within marine mammal communities across the Atlantic Ocean (Fig. 1 and Supplementary Table S1): western Ireland^31^, northern France^31^, the southern North Sea^32^, north-western Spain^33^, Mauritania^34^, northern Brazil^35^, southern Brazil^36^ and southern Argentina^37,38^. Additionally, the stable isotope ratios of C and N in bone samples of marine mammals from Uruguay have been analyzed for this study (Fig. 1 and Supplementary Table S1). Baleen whales, beaked whales (Ziphiidae), Risso’s dolphin *Grampus griseus*, pilot whales *Globicephala spp.* and southern elephant seals *Mirounga leonina* were not included in this study (Supplementary Table S1) because they are either filter feeders (baleen whales) or deep diving suction feeders^12–14^ and those feeding modes impose their own biomechanical and physiological constrains in association to a huge body mass^16,17^. Killer whales have also been excluded (Supplementary Table S1) because they are often involved in long distance migrations between areas differing in their isotopic baselines and therefore may show values which are not representative of the sampling location^39,40^. Conversely, the South American sea lion *Otaria byronia* has been included in this study, despite being considered a suction feeder by Kienle and Berta^13^ on the basis of its vaulted palate. It should be noted, however, that the diet of South American sea lions is dominated by fish, like that of other generalist pinnipeds^19^, while bivalves dominate the diet of truly specialized suction feeding pinnipeds^17,19^. Direct observation has revealed that generalist pinnipeds combine suction and biting feeding^17^ and hence, excluding the South American sea lion from the current study would be premature in the absence of direct observations confirming the prevalence of suction feeding.

### Stable isotope analysis of samples from Uruguay

Bone samples were collected from 147 skulls of eight marine mammal species (Supplementary Table S1): two otariid species (South American sea lion *Otaria byronia* and South American fur seal *Arctocephalus australis*) and six odontocete species, the pontoporiid franciscana dolphin *Pontoporia blainvillei*, the phocoenid Burmeister’s porpoise *Phocoena spinipinnis*, and four delphinids (common dolphin *Delphinus delphis*, Fraser's dolphin *Lagenodelphis hosei*, false killer whale *Pseudorca crassidens* and bottlenose dolphin *Tursiops truncatus*). All of them had been found stranded dead or incidentally caught by fishermen along the Uruguayan coastline between 1973 and 2016.

The bone samples of pinnipeds and cetaceans used for the isotopic analysis (C and N) consisted, respectively, of a small fragment of bone from the nasal cavity (turbinate bone) or the maxilla. All the skulls from South American sea lion, South American fur seal and franciscana dolphin were considered to belong to adult or physically mature specimens (see Drago et al*.*^41,42^ for details on age determination). For the remaining species, although the age and standard length of the individuals were unknown, the condylobasal length of each skull was measured to ensure that only specimens of similar body size were included and thus avoid age-related bias.

In the laboratory, bone samples were cleaned and processed as described in Drago et al*.*^41,42^. Approximately 1 mg of bone was weighed into tin capsules and analyzed by elemental analysis-isotope ratio mass spectrometry, specifically by means of a model FlashEA 1112 elemental analyzer (Thermo Fisher Scientific, Milan, Italy) coupled with a Delta C isotope ratio mass spectrometer (ThermoFinnigan, Bremen, Germany). All analyses were performed at the Centres Cientifics i Tecnològics of the University of Barcelona, Spain.

Stable isotope abundances are expressed in delta (δ) notation, with relative variations of stable isotope ratios expressed in per mil (‰) deviations from predefined international standards, and they were calculated as:

$$\delta^{j} {\text{X}} = \left[ {\left( {^{j} {\text{X}}/^{i} {\text{X}}} \right)_{{{\text{sample}}}} /\left( {^{j} {\text{X}}/^{i} {\text{X}}} \right)_{{{\text{standard}}}} } \right] - 1$$

where ^j^X is the heavier isotope (^13^C or ^15^N), and ^i^X is the lighter isotope (^12^C or ^14^N) in the analytical sample and international measurement standard^43^; international standards were the Vienna Pee Dee Belemnite (VPDB) calcium carbonate for the δ^13^C value and atmospheric nitrogen for the δ^15^N value. However, secondary isotopic reference materials given by the International Atomic Energy Agency (IAEA, Vienna, Austria), were used for calibration at a precision of 0.05 ‰ for carbon and 0.02 ‰ for nitrogen. The raw data were normalized by the multipoint normalization method based on linear regression^44^. Furthermore, we also quantified the carbon to nitrogen (C/N) atomic ratio of each analyzed sample as a control or proxy for the data quality of the bone collagen^45^. It ranged from 2.8 to 3.9, agreeing with the theoretical range that characterized unaltered proteins^45^.

### The Suess effect correction

During the last three centuries, the content of ^13^C-depleted in atmospheric CO_2_ has been increasing rapidly due largely to burning of fossil fuel. This phenomenon, invoked by different authors to explain the decline of δ^13^C values in several species, is called the Suess effect^46^. This effect needs to be taken into account to allow the comparison of the δ^13^C values from specimens from different periods^47^. Accordingly, bone δ^13^C values were corrected over time by applying the Suess effect correction factor computed by Verburg^47^. However, because the oceanic ^13^C Suess effect follows the atmospheric ^13^C Suess effect record with a roughly decadal lag^48,49^, the Verburg equation was modified in the present study to consider the 10 years lag existing for isotopic equilibration between atmospheric CO_2_ and oceanic aqueous CO_2_. Thus, the oceanic Suess effect correction factor was calculated as:

$$\begin{aligned} & 7.7738118 \, * \, 10^{ - 16} *\left( {Y - 10} \right)^{6} {-}1.2222044*10^{ - 11} *\left( {Y - 10} \right)^{5} \\ & \quad + 7.1612441*10^{ - 8} *\left( {Y - 10} \right)^{4} {-}2.1017147*10^{ - 4} *\left( {Y - 10} \right)^{3} \\ & \quad + 3.3316112*10^{ - 1} *\left( {Y - 10} \right)^{2} {-}273.715025 \, *\left( {Y - 10} \right) + 91703.261 \\ \end{aligned}$$

where *Y* is the year when the specimens were found stranded dead or incidentally caught by fishermen. The Suess corrected δ^13^C values (indicated with δ^13^C_cor_; see Supplementary Table S1) were referenced to the year 2007 for samples from Mauritania and to 2016 for those from Uruguay.

### Body weight, skull morphology and mouth gape measurement

The average body weight of each species was compiled from Wilson and Mittermeier ^2^, accounting for sexual dimorphism in pinnipeds (Table 1). Each odontocete species was classified as longirostrine, brevirostrine or intermediate according to McCurry et al*.*^14,15^ (Table 1). The palate breadth after postcanine 4 in pinniped and palate breadth between preorbital notches in cetaceans were used to assess mouth gape in the specimens from Mauritania studied by Pinela et al*.*^34^ and the specimens from Uruguay reported here. Palate breadth was measured in balanced samples of both sexes for the South American sea lion and the South American fur seal, as they exhibit considerable sexual dimorphism. The specimens from Mauritania (collected along the coastline between 1992 and 2007) belong to the scientific collection of the Faculty of Biology of the University of Barcelona (Spain) and those from Uruguay to the scientific collection of the National Museum of Natural History and the Faculty of Sciences of the University of the Republic at Montevideo (Uruguay).

**Table 1:** Average body weight and skull morphology classification for each marine mammal species. *n*: number of populations considered for each species.

Common name	Scientific name	Body weight (kg)	Skull morphology	*n*
**Cetaceans**				
Commerson’s dolphin	*Cephalorhynchus commersonii*	50	Brevirostrine	1
False killer whale	*Pseudorca crassidens*	1600	Brevirostrine	4
Fraser's dolphin	*Lagenodelphis hosei*	185	Brevirostrine	3
Southern right whale dolphin	*Lissodelphis peronii*	60	Brevirostrine	1
Spectacled porpoise	*Phocoena dioptrica*	140	Brevirostrine	1
Harbor porpoise	*Phocoena phocoena*	60	Brevirostrine	5
Burmeister’s porpoise	*Phocoena spinipinnis*	80	Brevirostrine	2
Atlantic white-sided dolphin	*Lagenorhynchus acutus*	170	Intermediate	2
White-beaked dolphin	*Lagenorhynchus albirostris*	275	Intermediate	3
Peale’s dolphin	*Lagenorhynchus australis*	115	Intermediate	1
Hourglass dolphin	*Lagenorhynchus cruciger*	100	Intermediate	1
Guiana dolphin	*Sotalia guianensis*	40	Intermediate	2
Bottlenose dolphin	*Tursiops truncatus*	175	Intermediate	5
Common dolphin	*Delphinus delphis*	95	Longirostrine	7
Franciscana dolphin	*Pontoporia blainvillei*	40	Longirostrine	1
Pantropical spotted dolphin	*Stenella attenuata*	120	Longirostrine	2
Striped dolphin	*Stenella coeruleoalba*	80	Longirostrine	3
Atlantic spotted dolphin	*Stenella frontalis*	105	Longirostrine	2
Spinner dolphin	*Stenella longirostris*	75	Longirostrine	1
Atlantic humpback dolphin	*Sousa teuszii*	100	Longirostrine	1
Rough-toothed dolphin	*Steno bredanensis*	120	Longirostrine	2
**Pinnipeds**				
South American fur seal	*Arctocephalus australis*	110		2
South American sea lion	*Otaria byronia*	225		2
Gray seal	*Halichoerus grypus*	170		2
Mediterranean monk seals	*Monachus monachus*	270		1
Harbor seal	*Phoca vitulina*	97		1

### Data analyses

Studies available in the literature included results from a variety of tissues (skin, muscle or bone) which are known to differ in their turnover ratios and trophic discrimination factors^50^. Taking this into account, the comparison of the isotopic niches of species from the same region was made always using the same tissue. Also, because baseline isotope ratios vary regionally^51^, a direct comparison of stable isotope ratios across regions was not attempted, even when the same tissue had been analysed. Accordingly, the comparison of isotopic niches across communities was based on the relative position of each species over the δ^13^C and the δ^15^N ranges of their communities (Supplementary Table S1), expressed as percentage within each range: 100% for inshore species and 0% for offshore species; 100% for the species with the highest tropic level and 0% for the species with the lowest trophic level. Spearman’s ρ correlation coefficient was used to evaluate the relationship between body weight and the relative position of each species along the δ^13^C and δ^15^N ranges.

Studies in the literature reported the average and the standard deviation of the δ^13^C and δ^15^N values of each species, but did not detail the values of each individual; this prevented a more detailed analysis on niche overlap in many cases. Individual δ^13^C and δ^15^N values were available only for marine mammal species from Uruguay, whose specimens were sampled in the present study, and for some of the specimens from Mauritania reported in Pinela et al*.*^34^ (Supplementary Table S1). Therefore, Stable Isotope Bayesian Ellipses in R (SIBER)^28^ was used to estimate the isotopic niche width of marine mammal species from only those two localities (Uruguay and Mauritania). This allowed us to assess whether overall isotopic width of niches, overlap and trophic relationships (i.e., the relative positions of species niches in the isotopic space) among the marine mammal species was different within each considered community. We used standard ellipse areas corrected for small sample size (SEA_C_) to plot the isotopic niche of each species within the isotopic space and to calculate the overlap among species. We also calculated the Bayesian standard ellipse areas (SEA_B_) to obtain an unbiased estimate of the isotopic niche width with credibility intervals. We used these two approaches because they are complementary each other^28^.

We also compared the palate breadth among species within each considered marine mammal community (Uruguay and Mauritania) using one-way ANOVA, followed by a Scheffé post-hoc test. Spearman’s ρ correlation coefficient was used to evaluate the relationship between δ^15^N values and palate breadth, and between δ^15^N values and body mass within each considered marine mammal community (Uruguay and Mauritania). The same procedure was used to determine whether a relation existed between the isotopic niche width (estimated through SEA_B_) and palate breadth within each considered community. Niche similarity was assessed by computing the Euclidean distance between the centroids of each species in the δ^13^C–δ^15^N bi-plot space, whereas morphologic similarity was assessed by computing the Euclidean distance between species within the morphospace as defined by palate breadth. Isotopic niche and morphologic similarities were compared in each marine mammal community (Uruguay and Mauritania) using the Mantel test.

Prior to statistical analyses, normality was tested by means of the Lilliefors test, and homoscedasticity by means of the Levene test. Data are always shown as mean ± standard deviation (SD) unless otherwise stated. All statistical analyses were carried out using the free software R^52^, and all codes for SIBER analyses are contained in the package SIBER^28^.

## Results

We compiled the δ^13^C and δ^15^N values of 26 species of marine mammals from 9 localities, each of them including at least 4 species (Fig. 1 and Supplementary Table S1). This resulted in a data set including 58 populations (species x locality; Table 1). Each of the following species was sampled from only a single locality: Commerson’s dolphins *Cephalorhynchus commersonii*, hourglass dolphins *Lagenorhynchus cruciger*, Peale’s dolphins *Lagenorhynchus australis*, Mediterranean monk seals *Monachus monachus*, franciscana dolphins, spectacled porpoises *Phocoena dioptrica*, harbor seals *Phoca vitulina* and Atlantic humpback dolphins *Sousa teuszii*. Sample size was at least 5 for each of those species. The remaining 18 species were sampled from at least two distinct localities. Usually more than 5 specimens were analyzed at each locality, except for grey seals *Halichoerus grypus*, false killer whales and Fraser’s dolphins at some localities (Supplementary Table S1).

**Figure 2:**
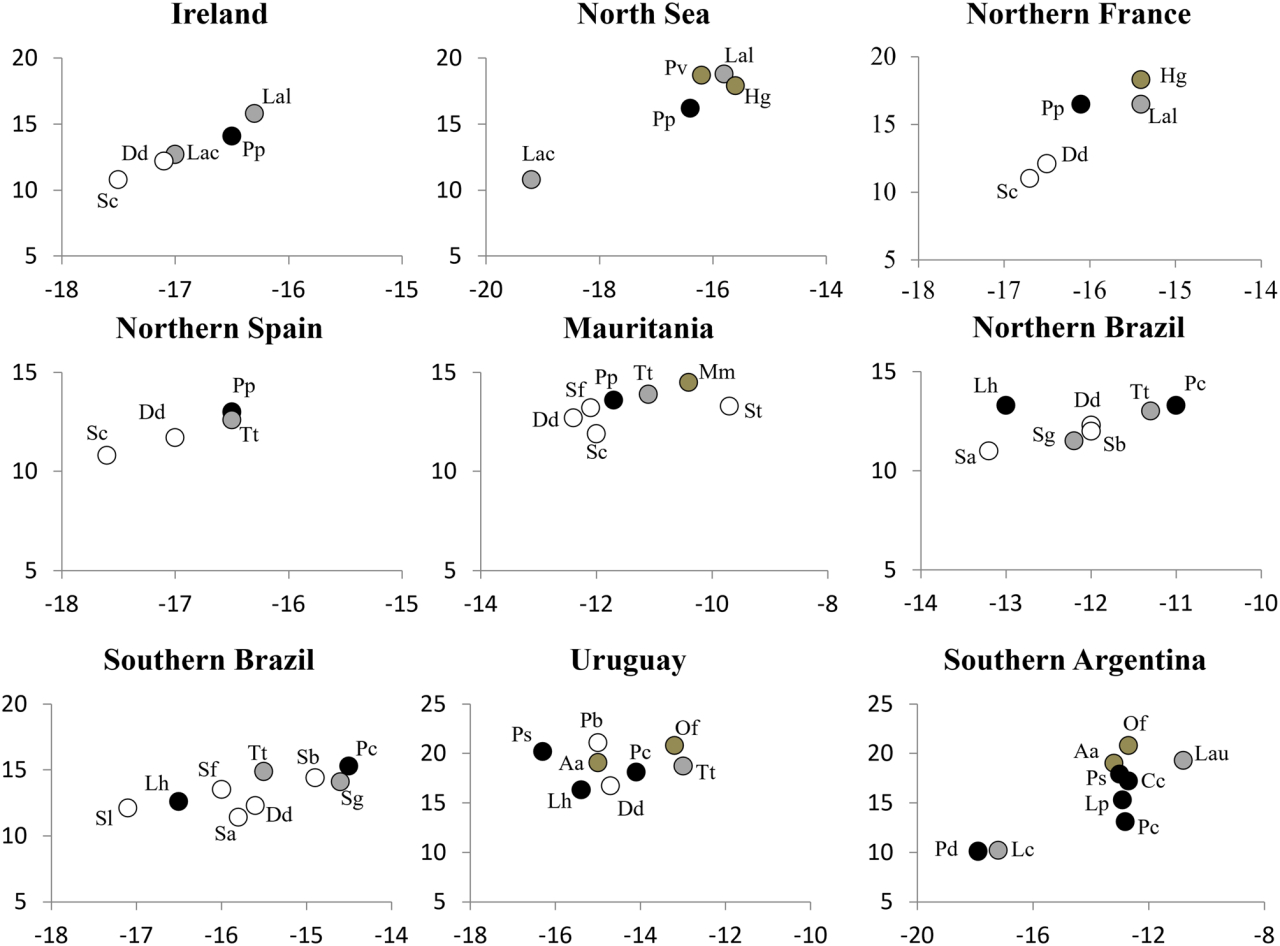
Topology of biting marine mammals in the isospace. Species: common dolphin (Dd), white-beaked dolphin (Lal), Atlantic white-sided dolphin (Lac), harbor porpoise (Pp), striped dolphin (Sc), harbor seal (Pv), gray seal (Hg), bottlenose dolphin (Tt), Mediterranean monk seal (Mm), Atlantic humpback dolphin (St), Atlantic spotted dolphin (Sf), false killer whale (Pc), rough-toothed dolphin (Sb), Guiana dolphin (Sg), Fraser's dolphin (Lh), pantropical spotted dolphin (Sa), spinner dolphin (Sl), franciscana dolphin (Pb), Burmeister's porpoise (Ps), South American sea lion (Of), South American fur seal (Aa), southern right whale dolphin (Lp), spectacled porpoise (Pd), hourglass dolphin (Lc), Peale’s dolphin (Lau), Commerson’s dolphin (Cc). Morphological groups: pinnipeds (brown dots) and brevirostrine (black dots), intermediate (gray dots) and longirostrine (white dots) odontocetes. Plot axes: X = δ^13^C (‰); Y = δ^15^N (‰).

Most species had highly consistent positions within the regional δ^13^C–δ^15^N isospaces. Common dolphins, striped dolphins *Stenella coeruleoalba*, Atlantic spotted dolphins *Stenella frontalis*, pantropical spotted dolphins *Stenella attenuate* and Atlantic white-sided dolphins *Lagenorhynchus acutus* were always highly depleted in both ^13^C and ^15^N isotopes within their communities, thus revealing consistent offshore foraging at a low trophic position (Fig. 2). Hourglass dolphins and spectacled porpoises had a similar position in the isoscape of southern Argentina (Fig. 2). Conversely, white-beaked dolphins, bottlenose dolphins, grey seals and South America sea lions were consistently enriched in both ^13^C and ^15^N isotopes within their communities, thus revealing inshore foraging at high trophic positions (Fig. 2). Harbor seals and Mediterranean monk seals were also enriched in both ^13^C and ^15^N isotopes within their communities and hence had isotopic niches similar to those of pinnipeds reported above. The same was also true for Peale’s dolphins off southern Argentina (Fig. 2). South American fur seals were consistently depleted in both ^13^C and ^15^N isotopes compared to sympatric South American sea lions, thus revealing a less inshore habitat and a lower trophic position (Fig. 2).

Other species had highly consistent positions along one of the axes of the isospace and more variable positions in the other axis. Harbor porpoise *Phocoena phocoena* and Burmeister’s porpoise where consistently enriched in ^15^N within their communities in the eastern North Atlantic Ocean and the western South Atlantic Ocean, respectively, but their positions along the δ^13^C axis were more variable (Fig. 2). At most localities, harbor porpoises were depleted in ^13^C compared to sympatric white-beaked dolphins *Lagenorhynchus albirostris* or bottlenose dolphins, but they did not differ in δ^13^C values from bottlenose dolphins off northern Spain (Fig. 2). More strikingly, the δ^13^C values of Burmeister’s porpoises suggested offshore foraging in Uruguay and inshore foraging off southern Argentina (Fig. 2). Conversely, false killer whales were consistently enriched in ^13^C from northern Brazil to southern Argentina, but their positions along the δ^15^N axis were highly variable and suggestive of a decreasing trophic position at higher latitudes (Fig. 2). Fraser’s dolphins, on the contrary, were consistently depleted in ^13^C everywhere, thus revealing offshore habitats from northern Brazil to Uruguay, but their position along the δ^15^N axis dropped in Southern Brazil and Uruguay (Fig. 2). Guiana dolphins *Sotalia guianensis* and rough-toothed dolphins *Steno bredanensis* had similar values of both δ^13^C and δ^15^N in the two localities where they co-occurred, but their topologies within the community were variable (Fig. 2).

Finally, two species reported from only one locality each had unusual isotopic niches. Atlantic humpback dolphins were the most inshore species in Mauritania and franciscana dolphins were among top predators in Uruguay (Fig. 2).

The range of the δ^15^N values in the 9 communities of marine mammals considered increased significantly with latitude (r = 0.712, *P* = 0.031, n = 9), from 2.3 ‰ in northern Brazil to 10.7 ‰ in southern Argentina (Fig. 2). The variation range of δ^13^C values was narrower (1.1 to 5.2 ‰) and was unrelated to latitude (*P* = 0.590, n = 9; Fig. 2). This regional variability, combined with the diversity of tissues analyzed (Supplementary Table S1), hindered the direct comparison of stable isotope ratios across regions.

A weak, statistically significant and positive correlation was observed between body size and the relative position of populations both along the δ^13^C axis (Spearman’s ρ = 0.321, *P* = 0.014, n = 58) and the δ^15^N axis (Spearman’s ρ = 0.276, *P* = 0.036, n = 58). These correlations were mainly driven by the large body size, trophic position and inshore habitats of false killer whales, white-beaked dolphins, Mediterranean monk seals and South American sea lions. A more robust, statistically significant and positive correlation was observed between the relative position of populations along the δ^13^C and δ^15^N axes (Spearman’s ρ = 0.674, *P* < 0.001, n = 58), thus confirming that inshore species had a higher trophic position than offshore species.

**Figure 3:**
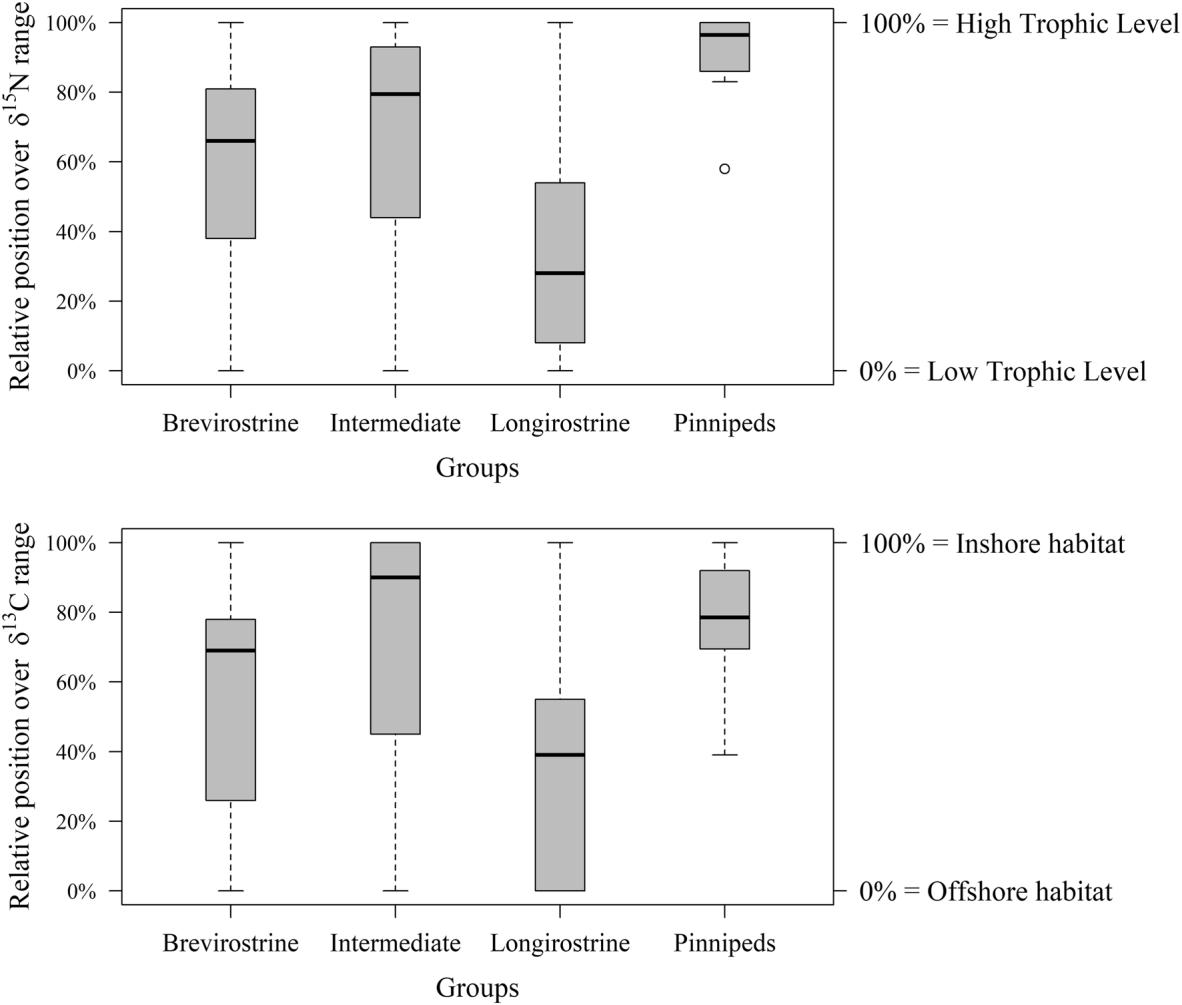
Boxplots summarizing the distribution of the relative position of populations of brevirostrine, intermediate and longirostrine biting odontocetes and pinnipeds along the δ^15^N and δ^13^C ranges. Boxes represent first and third quartiles, lines the median and whiskers the 95% confidence interval.

The populations of the four morphological groups differed significantly in their distributions along the δ^13^C and δ^15^N axes (Fig. 3; Kruskall-Wallis test; δ^13^C: Chi-square = 11.465, df = 3, n = 58, *P* = 0.009; δ^15^N: Chi-square = 17.753, df = 3, n = 58, *P* < 0.001). Populations of longirostrine odontocetes usually were highly depleted in ^13^C except those of Atlantic humpback dolphins and rough-toothed dolphins, and one population of Guiana dolphins (Figs. 2 and 3). They were also highly depleted in ^15^N, except all franciscana dolphins, one population of Guiana dolphins and one population of rough-toothed dolphins (Figs. 2 and 3). Brevirostrine odontocetes were usually enriched in ^13^C except the three populations of Fraser’s dolphin, the spectacled porpoise *Phocoena dioptrica* and one population of the Burmeister’s porpoise (Figs. 2 and 3). Likewise, brevirostrine odontocetes were usually enriched in ^15^N except two populations of Fraser’s dolphins and two populations of false killer whales. Intermediate odontocetes were consistently enriched in both ^13^C and ^15^N (Figs. 2 and 3), except hourglass dolphins which were extremely depleted in both. Finally, pinnipeds were highly enriched in both ^13^C and ^15^N except one population of South American fur seals (Figs. 2 and 3).

**Figure 4:**
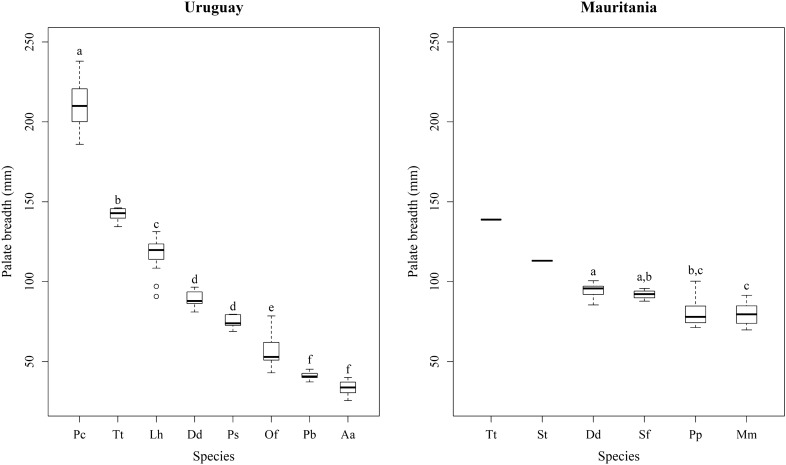
Boxplots of the palate breadth of the marine mammal species from Uruguay and Mauritania. Species, within each community, with different superscript (lower case letters) are statistically different in their mean values according to the Scheffé post-hoc test following nested ANOVA. Species without superscript were not included in the statistical analyses due to the small sample size (< 4 specimens). Boxes represent the first and third quartile, lines the median and whiskers 95% confidence interval. Sample size for species: false killer whale (Pc; n = 11), bottlenose dolphin (Tt; n = 5 Uruguay; n = 1 Mauritania), Fraser's dolphin (Lh; n = 30), common dolphin (Dd; n = 6 Uruguay; n = 15 Mauritania), Burmeister's porpoise (Ps; n = 5), South American sea lion (Of; n = 29), franciscana dolphin (Pb; n = 25), South American fur seal (Aa; n = 33), Atlantic humpback dolphin (St; n = 2), Atlantic spotted dolphin (Sf; n = 4), harbor porpoise (Pp; n = 12) and Mediterranean monk seal (Mm; n = 12).

Statistically significant differences existed in the palate breadth among species within each marine mammal community. For the Uruguayan community (ANOVA: F_7,136_ = 885.54, *P* < 0.001), post-hoc tests revealed that false killer whales, followed by bottlenose dolphins, had the broadest palate; franciscana dolphins and South American fur seals had the narrowest one (Fig. 4). Common dolphins and Burmeister's porpoises did not differ in palate breadth, which in these species was intermediate between that of Fraser's dolphins and South American sea lions (Fig. 4). For the Mauritanian community (ANOVA: F_3,39_ = 14.04, *P* < 0.001), post-hoc tests revealed that common dolphins had a broader palate than Mediterranean monk seals, whereas Atlantic spotted dolphins and harbor porpoises showed intermediate values in palate breadth (Fig. 4). Bottlenose dolphins, followed by Atlantic humpback dolphins, had the broadest palate but they were not included in the statistical analyses due to their small sample size (Fig. 4).

**Figure 5:**
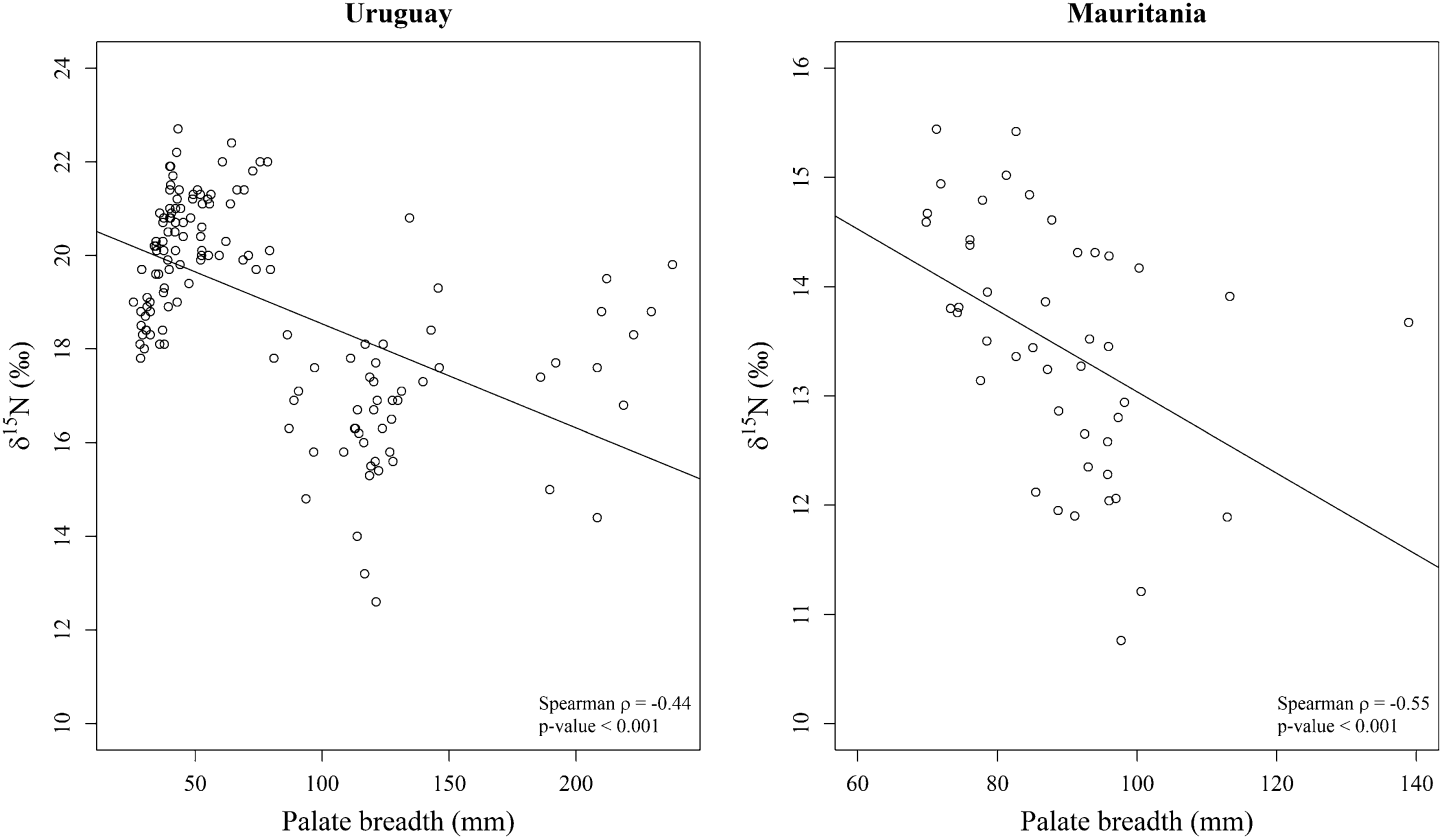
Relationship between δ^15^N values and breadth of palate in the marine mammals from Uruguay and Mauritania.

The δ^15^N value was negatively correlated with palate breadth both in the Uruguay and Mauritania communities (Fig. 5). On the contrary, δ^15^N value and body mass were uncorrelated in either the Uruguay (Spearman’s ρ = − 0.48; *P* = 0.24) and the Mauritania (Spearman’s ρ = − 0.08; *P* = 0.91) communities. These results suggest that trophic level decreased with palate breadth in both communities without any effect of the body mass.

**Figure 6:**
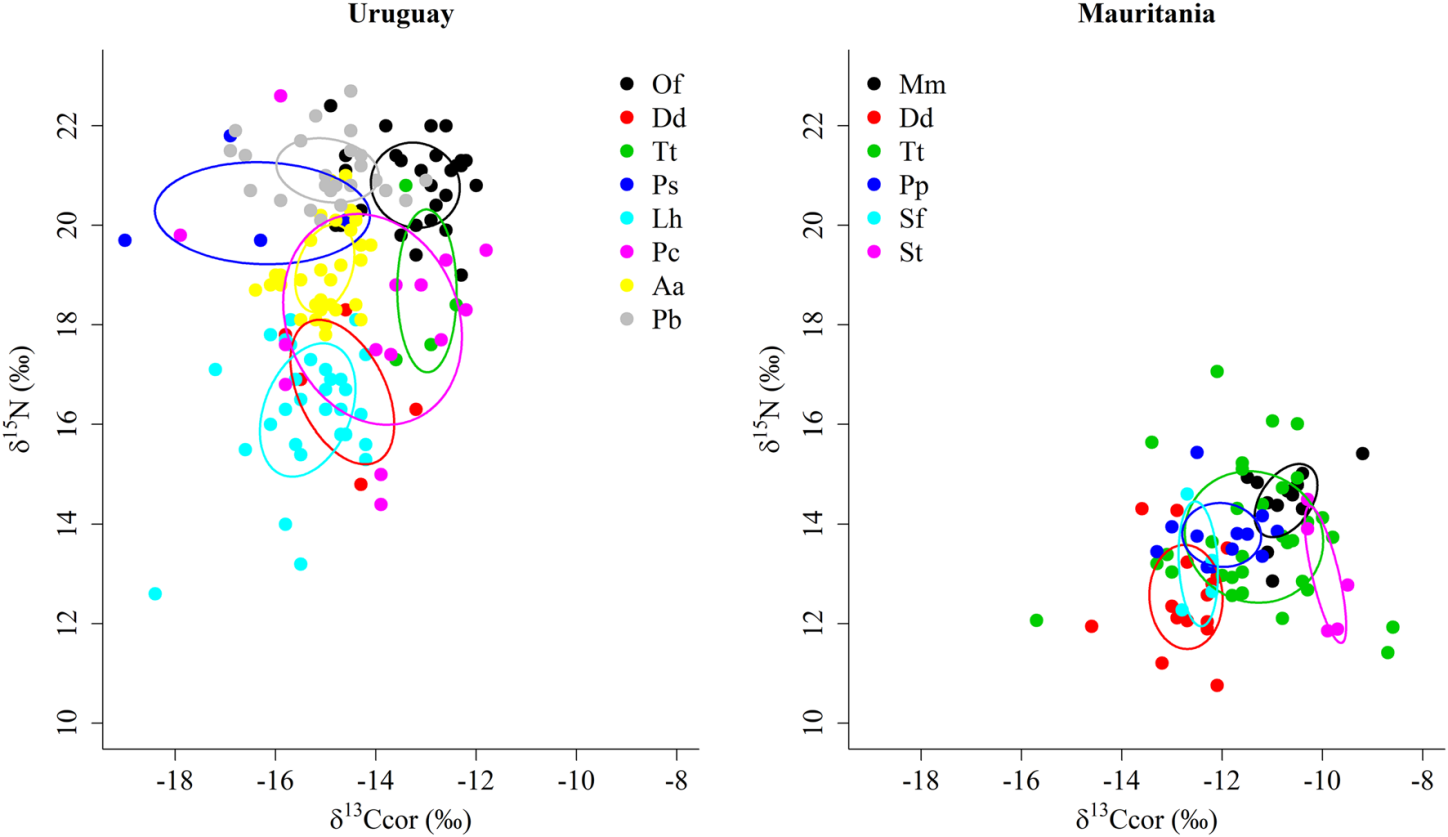
Isotopic niche areas calculated with SEA_C_ (see Supplementary Table S2 for the ellipse area, credibility interval and overlap area values) for the marine mammal species from Uruguay and Mauritania. Species: South American sea lion (Of), common dolphin (Dd), bottlenose dolphin (Tt), Burmeister's porpoise (Ps), Fraser's dolphin (Lh), false killer whale (Pc), South American fur seal (Aa), franciscana dolphin (Pb), Mediterranean monk seal (Mm), harbor porpoise (Pp), Atlantic spotted dolphin (Sf) and Atlantic humpback dolphin (St). δ^13^C_cor_: values corrected for Suess effect shifts (see original data and sample size in Supplementary Table S1).

**Figure 7:**
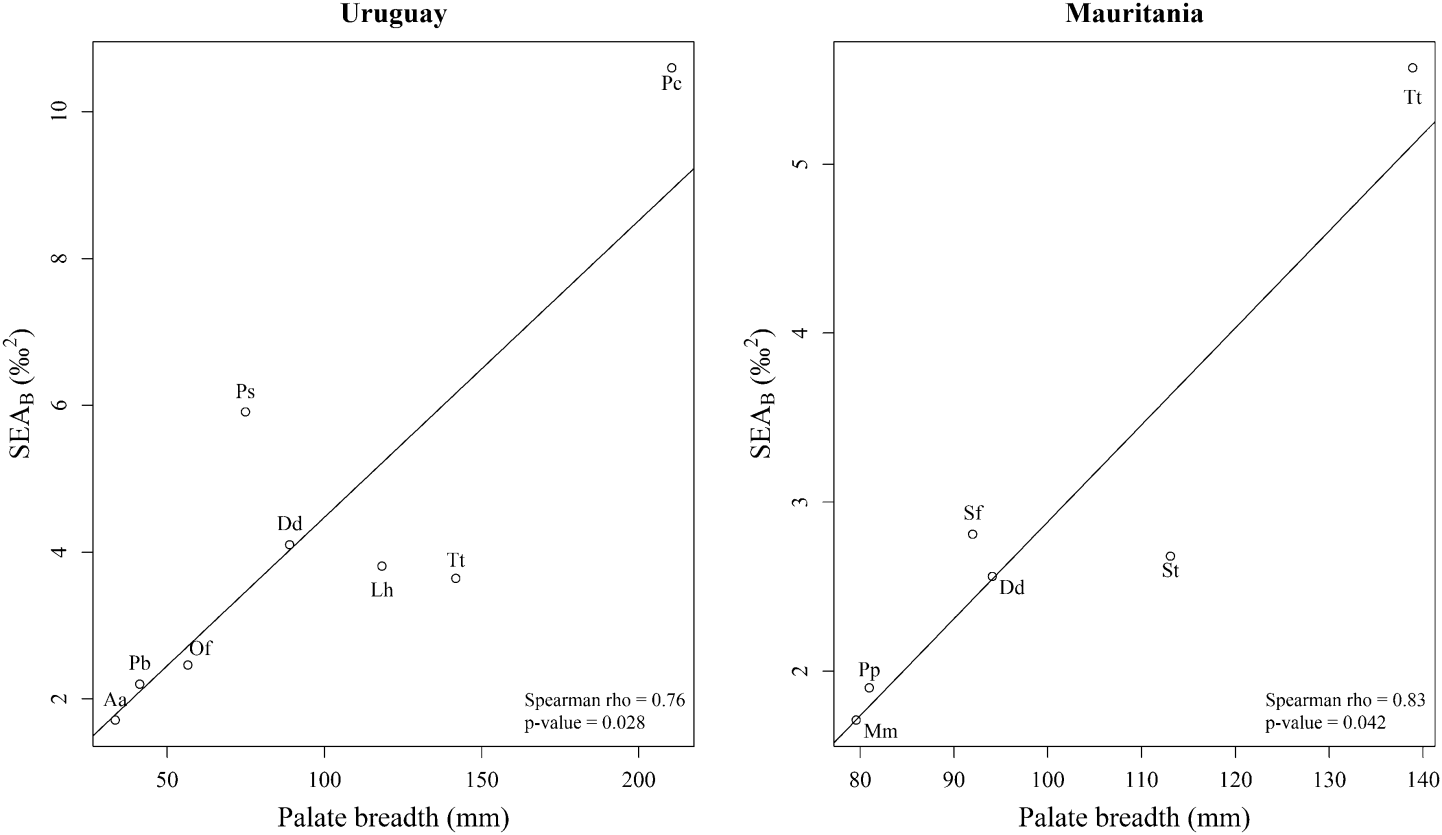
Relationship between the isotopic niche width (estimated by SEA_B_; see Supplementary Table S2 for the ellipse area values) and palate breadth in the marine mammals from Uruguay and Mauritania. Species: South American sea lion (Of), common dolphin (Dd), bottlenose dolphin (Tt), Burmeister's porpoise (Ps), Fraser's dolphin (Lh), false killer whale (Pc), South American fur seal (Aa), franciscana dolphin (Pb), Mediterranean monk seal (Mm), harbor porpoise (Pp), Atlantic spotted dolphin (Sf) and Atlantic humpback dolphin (St).

On the other hand, morphologic similarity and niche similarity were also uncorrelated for either the Uruguay (Mantel test: *P* = 0.64) and the Mauritania (Mantel test: *P* = 0.55) communities. That was so because in both communities the isotopic niches of morphologically dissimilar species (i.e., species with dissimilar palate breadth) overlapped (Fig. 6). In the community from Uruguay, this was especially the case for false killer whales and common dolphins, bottlenose dolphins, Fraser's dolphins and South American fur seals. Indeed, the palate breadth and the estimated ellipse area of false killer whales were the largest of all the considered species and the isotopic niche overlapped widely with that of the aforementioned species (Figs. 4 and 6, and Supplementary Table S2). Conversely, morphologically more similar species (i.e., species with similar palate breadth), such as South American fur seals and franciscana dolphins or as common dolphins and Burmeister's porpoises, did not overlap at all in their isotopic niches (Figs. 4 and 6, and Supplementary Table S2). Similar patterns were observed in Mauritania, particularly in the case of bottlenose dolphins and all the other considered species from that community. The palate breadth and the estimated ellipse area of bottlenose dolphins were larger than those of the other species and the isotopic niche overlapped widely (Figs. 4 and 6, and Supplementary Table S2). In conclusion, the detailed analysis of the marine mammal communities of Mauritania and Uruguay revealed low overlap between the isotopic niches of most pairs of sympatric species, although in both regions the odontocete species with the broadest mouth gape (the bottlenose dolphin off Mauritania and the false killer whale off Uruguay) largely overlapped with several other species. It is worth noting that patterns of overlap were similar in both regions despite the much narrower δ^15^N range in Mauritania. Likewise, a significant and positive relationship was observed in both regions between the isotopic niche width and the palate breadth (Fig. 7).

## Discussion

The results reported here revealed highly consistent isotopic niches for most species of biting odontocetes and pinnipeds and a conserved topology of their community within the isospace across the Atlantic Ocean. They also revealed body size as a poor predictor of the isotopic niche in biting marine mammals and a modest role of skull morphology on determining it. Furthermore, pinnipeds exhibited a much lower diversity of isotopic niches than odontocetes, which agrees with a much lower variability in skull morphology^12–15^.

It should be noted that these results were derived from the analysis of a data set including information on the skull morphology, body size and C and N stable isotope ratios of 26 species and 58 populations, hence including most of the variability existing in the Atlantic Ocean and offering a high degree of generalization. On the contrary, the inverse relationship between trophic position and palate breadth and the positive relationship between palate breadth and the width of the isotopic niche were derived from a more limited data set and hence may not be as general. It does not necessarily include the whole diversity of skull morphology, body size and diets reported for pinnipeds and odontocetes worldwide, which limits the generalization of the conclusions from the present study. This limitation is even more severe when considering the relationship between palate breadth, trophic position and the breadth of the isotopic niche, as the results reported arouse from the study of only two localities. Further research, including other species and regions, is required to confirm the generality of the conclusions reported here. Another relevant issue is the impossibility of direct comparison of stable isotope ratios from different studies, because of methodological and ecological factors. On the one hand, the diversity of tissues analyzed in the literature and the consistent differences in the turnover ratios and trophic discrimination factors between tissues^29,50,53,54^ prevent direct comparison between regions and studies. On the other hand, variation in isotopic baselines (i.e., the stable isotope ratios in primary producers) due to differences in biogeochemical processes^29,55^ makes even more challenging any direct comparison. Finally, the negative correlation between trophic discrimination factors and water temperature reported for poikilothermic species^56,57^ apparently results in the increasing range of δ^15^N values observed in marine communities located at high latitudes^58^. This effect undoubtedly propagates to their predators and explains why the δ^15^N range is much larger in the North Sea and southern Argentina than in Brazil or Mauritania. Thus, in the absence of an adequate local reference, calculating the actual trophic position of each species is impracticable. For this reason, we used the relative position along the δ^13^C and δ^15^N ranges as a coarse proxy of trophic niche.

Independently of these limitations, most species had rather consistent isotopic niches, particularly common dolphins, striped dolphins, spotted dolphins, Atlantic white-sided dolphins, white-beaked dolphins, bottlenose dolphins, grey seals, South American sea lions and South America fur seals. Previous research based on stomach content and scat analyses revealed that the prey species varies between areas but also that the diet consistently includes the same type of prey (see Supplementary Table S3). For instance, common dolphins tend to consume small neritic fishes, striped and spotted dolphins to consume small oceanic fishes and grey seals, South American sea lions and South America fur seals tend to consume small and medium size demersal fishes. It is worth noting that inshore and offshore ecotypes have been previously described for bottlenose dolphins in many parts of the world^59^, including in the northeastern^60^ and southwestern Atlantic Ocean^61^. Despite such supposed intraspecific variability, oceanic prey are uncommonly reported in the diet of bottlenose dolphins (see Supplementary Table S3), which explains their consistent position in the isospace when compared to other species. This suggests that most studies so far reported on this species have been conducted on individuals from the inshore ecotype, which would have higher chances to be washed ashore after death and therefore of being sampled by stranding programs, or which would be more accessible to researchers conducting biopsy sampling.

Other species had a consistent trophic position or a consistent habitat, but not both of them. Atlantic and Burmeister’s porpoises were consistent in their trophic position, but not in habitat use, whereas the opposite was true for false killer whales and Fraser’s dolphins, which emerged consistently as inshore and offshore predators, respectively. Regarding false killer whales, they are often considered to have offshore distribution, but oxygen isotope ratios had previously confirmed inshore foraging off Uruguay^62^. C isotope ratios confirm this. Also, in this species the trophic position drops at high latitudes, where it appears to largely rely on squids^63^.

As a result of the above regularities, the 9 communities shared a similar topology in the δ^13^C–δ^15^N isospace. Everywhere, coastal habitats supported at least one cetacean species with an intermediate skull morphology and a high trophic position: bottlenose dolphins in warm temperate and tropical regions, white-beaked dolphins in cold temperate regions of the eastern North Atlantic Ocean, and Peale’s dolphins in the cold temperate regions of the western South Atlantic Ocean. Where other species of coastal dolphins species existed, they differed largely in body size and skull morphology: intermediate bottlenose dolphins and longirostrine Atlantic humpback dolphins in Mauritania (175 and 100 kg), brevirostrine false killer whales, intermediate bottlenose dolphins and longirostrine Guiana dolphins in northern and southern Brazil (1600, 175 and 40 kg), and brevirostrine false killer whales, intermediate bottlenose dolphins and longirostrine franciscana dolphins in Uruguay (1600, 175 and 40 kg). Pinnipeds, always foraging at a high trophic position, inhabited also the coastal habitats of temperate regions of both hemispheres, as well as off subtropical Mauritania. Brevirostrine porpoises occurred in the temperate regions of both hemispheres and off Mauritania, and everywhere used less coastal habitats than the species of the preceding groups although had a similar, high trophic position. Finally, at least one species of longirostrine dolphin species occurred offshore everywhere, except in the southern North Sea and off Southern Argentina, where that niche was filled by a small species of the genus *Lagenorhynchus*. Where several species of longirostrine dolphins coexisted, common dolphins had consistently a higher trophic position than spotted or striped dolphins, except in southern Brazil.

Body size was correlated with trophic position only because a few species exceeded 200 kg and these were highly influential. Below that threshold of body weight, no correlation was observed and the small porpoises and the franciscana dolphin appeared among the top predators of their communities. Skull morphology is a better predictor of trophic niche, as longirostrine odontocetes have on average a lower trophic position than any other group. McCurry et al*.*^14^ argued that elongate and brevirostrine morphotypes that feed using biting prey capture strategies likely evolved as adaptations to exploit dietary resources at the lower and higher end of the prey size spectrum, respectively. Longirostrine biting feeding odontocetes typically consume small prey whereas brevirostrine biting feeding odontocetes typically consume medium-large prey. Longirostrine species will be able to resist lower loads during feeding, but would be able to move their snouts through the water at a faster speed to catch small prey.

However, a longirostrine morphology may not have necessary evolved to prey on small pelagic fishes and does not necessarily prevent from preying at a high trophic position. This is particularly true for franciscana dolphins *Pontoporia blainvillei*, whose diet is actually dominated by bottom-dwelling croackers (Family Sciaenidae) captured in coastal, turbid waters^2^. It should be noted that franciscana dolphins have a flexible neck and a skull morphology rather different from that of longirostrine delphinids^64^. Thus, functional diversity exists also within longirostrine cetaceans. When the franciscana dolphin is removed from the analysis, the remaining longirostrine odontocetes have always lower trophic positions than sympatric brevirostrine or intermediate odontocetes and pinnipeds, except in southern Brazil as a result of the effect in this location of Fraser’s dolphins, which are offshore predators foraging at a very low-level position. Likewise, the brevirostrine spectacled porpoises and, occasionally, the also brevirostrine Burmesiter’s porpoise, as well as the intermediate hourglass dolphin, rely heavily on offshore prey. This demonstrates that a longirostrine skull morphology is not necessary to inhabit pelagic, offshore habitats.

Traditionally, most pinnipeds and odontocetes were thought not to orally process their prey, except those species using grip and tear for the handling of warm-blooded prey^12^. However, there is increasing evidence that some species of pinnipeds and the bottlenose dolphin tear apart large prey by shaking and tearing^2,16,24^. The results reported here demonstrate that both in Uruguay and Mauritania palate breath is negatively correlated with trophic position. This is partially because pinnipeds with narrow palates have high trophic positions in both areas, but the correlation still stands when pinnipeds are removed from the analysis. There are at least two reasons for that unexpected pattern. First, longirostrine species often have broad palates, but show preference for consuming small prey well below their prey maximum, to minimize drag and optimize the capture of fast swimming fish. Second, species with broad mouths may consume a diversity of prey sizes^14^ and do not necessarily have a high trophic position. This suggests that species identity is probably more important than body size or skull morphology in determining the isotopic niche of biting marine mammals, although the generality of this conclusion is limited by sample size (two study sites, each with 1–2 pinniped and 5–6 cetacean species). Certainly, a broad diversity of skull shapes (from brevirostrine to longirostrine) and palate breadths (from 40 to 240 mm and from 70 to 140 mm) existed in each locality, but more study sites and a broader diversity of species should be included in future studies to further test the hypothesis that species identity is more relevant than body size or skull morphology in biting marine mammals.

Over the last three decades, the size-based analysis of food webs has largely contributed to provide generalizations regarding food web properties^65–72^. Such a conceptual framework assumes that species with a similar body size will have similar diets, and hence that the topology of species within the food webs will be largely determined by body size^72^. In this way, size-based analysis offers a mechanistic, highly reductionist approach that, when analysing complex food webs, allows to deal with a multitude of species whose body sizes span several orders of magnitude, from grams (e.g. zooplankton) to hundreds of kilograms (e.g. marine mammals). However, when considering species within the same order of magnitude, the relevance of differences in body size may decrease and other factors, such skull morphology, can be more relevant to determine resource use patterns^73–77^.

Indeed, the highly consistent isotopic niches across areas of the species studied here reveal rather narrow fundamental niches and invariant trophic positions across time and space, likely because of morphological constrains. Accordingly, the broad taxonomic diversity of the prey consumed by most biting marine mammal species should not be interpreted as evidence of trophic flexibility. Prey species are not totally interchangeable, something critical to keep in mind when assessing the indirect impact of fishing on biting marine mammal species. Likewise, each biting marine mammal species has a unique ecological niche, as revealed here, and hence their functional roles in marine food webs are not redundant. This should also be kept in mind when modelling the dynamics of marine food webs, because biting marine mammals should not be clumped for analysis in a single category. These considerations should be incorporated in ecosystem modelling exercises, where species are often clumped in groups for simplicity, often without proper justification.

## Supplementary Information

Supplementary Information.

## Data Availability

Data available from the University of Barcelona Digital Repository 10.34810/data126.
