# Supplementary material for: The isotopic niche of Atlantic, biting marine mammals and its relationship to skull morphology and body size
Source: Sci Rep. 2021 Jul 26;11:15147. doi: 10.1038/s41598-021-94610-w (PMC8313526; doi:10.1038/s41598-021-94610-w)
Supplement: Supplementary file 1 — Supplementary Information. [file 41598_2021_94610_MOESM1_ESM.pdf]

The isotopic niche of Atlantic, biting marine mammals and its relationship to skull morphology and body size

Massimiliano Drago, Marco Signaroli, Meica Valdivia, Enrique M. González, Asunción Borrell, Alex Aguilar, Luis Cardona.

SUPPLEMENTARY INFORMATION

SUPPLEMENTARY TABLES

Table S1. Mean and standard deviation of stable isotope values ($\delta^{13}C$ and $\delta^{15}N$) in sampled tissues of marine mammal species from North and South Atlantic Ocean. $\delta^{13}C_{cor}$: isotope values corrected for Suess effect shifts; $\%^{15}N$ and $\%^{13}C$: relative position of each species over the $\delta^{15}N$ and $\delta^{13}C$ ranges of their communities; $\Delta^{15}N$ and $\Delta^{13}C$: increase in the $\delta^{15}N$ and $\delta^{13}C$ values of each species with respect to the species with lower $\delta^{15}N$ (lowest trophic level) and $\delta^{13}C$ (most offshore habitat) value within the range of their community. The source of the stable isotope and the sample size for each specie (*n*) are also indicated.

Region/Locality	Species excluded	Species included	*n*	Tissue	$\delta^{15}N$ (‰)	$\delta^{13}C$(‰)	$\delta^{13}C_{cor}$(‰)	$\Delta^{15}N$ (‰)	$\%^{15}N$	$\Delta^{13}C$ (‰)	$\%^{13}C$	Source
**Eastern North Atlantic**												
Ireland		*Delphinus delphis*	14	Muscle	12.2 ± 1.0	-17.1 ± 0.4		1.4	28	0.4	33	[1]
		Lagenorhynchus acutus	4	Muscle	12.7 ± 0.5	-17.0 ± 0.5		1.9	38	0.5	42	
		Lagenorhynchus albirostris	3	Muscle	15.8 ± 2.3	-16.3 ± 0.3		5.0	100	1.2	100	
		Phocoena phocoena	7	Muscle	14.1 ± 1.6	-16.5 ± 0.7		3.3	66	1.0	83	
		Stenella coeruleoalba	3	Muscle	10.8 ± 0.6	-17.5 ± 0.1		0.0	0	0.0	0	
Northern France		*Delphinus delphis*	8	Muscle	12.1 ± 0.4	-16.5 ± 0.5		1.1	15	0.2	15	[1]
		Halichoerus grypus	1	Muscle	18.3	-15.4		7.3	100	1.3	100	
		Lagenorhynchus albirostris	1	Muscle	16.5	-15.4		5.5	75	1.3	100	
		Phocoena phocoena	4	Muscle	16.5 ± 2.4	-16.1 ± 0.6		5.5	75	0.6	46	
		Stenella coeruleoalba	3	Muscle	11.0 ± 1.8	-16.7 ± 0.4		0.0	0	0.0	0	
North Sea	*Balaenoptera physalus*	*Halichoerus grypus*	6	Muscle	17.9 ± 2.1	-15.6 ± 1.6		7.1	89	3.6	100	[2]
	Physeter macrocephalus	*Lagenorhynchus acutus*	2	Muscle	10.8 ± 0.4	-19.2 ± 0.1		0.0	0	0.0	0	
		Lagenorhynchus albirostris	7	Muscle	18.8 ± 1.1	-15.8 ± 0.7		8.0	100	3.4	94	
		Phocoena phocoena	46	Muscle	16.2 ± 1.6	-16.4 ± 1.6		5.4	68	2.8	78	
		Phoca vitulina	23	Muscle	18.7 ± 2.5	-16.2 ± 1.3		7.9	99	3.0	83	
Northern Spain	*Globicephala melas*	*Delphinus delphis*	114	Muscle	11.7 ± 0.6	-17.0 ± 0.5		1.1	50	0.6	55	[3]
		Phocoena phocoena	19	Muscle	13.2 ± 1.2	-16.5 ± 0.7		2.2	100	1.1	100	
		Stenella coeruleoalba	21	Muscle	10.8 ± 0.7	-16.6 ± 0.6		0.0	0	0.0	0	
		Tursiops truncatus	9	Muscle	12.6 ± 0.9	-16.5 ± 0.8		1.8	82	1.1	100	
Mauritania	*Balaenoptera acutorostrata*	*Delphinus sp.*	95	Bone	12.7 ± 0.8	-12.4 ± 0.8		0.8	31	0.0	0	[4]
	Globicephala macrorhynchus	*Monachus monachus*	12	Bone	14.5 ± 0.7	-10.4 ± 0.6		2.6	100	2.0	74	
	Globicephala melas	*Phocoena phocoena*	42	Bone	13.6 ± 0.8	-11.7 ± 0.9		1.7	65	0.7	26	
	Grampus griseus	*Sousa teuszii*	11	Bone	13.3 ± 1.3	-9.7 ± 0.9		1.4	56	2.7	100	
	Orcius orca	*Stenella coeruleoalba*	1	Bone	11.9	-12.0		0.0	0	0.4	0.15	
	Physeter macrocephalus	*Stenella frontalis*	4	Bone	13.2 ± 1.0	-12.1 ± 0.3		1.3	50	0.3	11	
		Tursiops truncatus	60	Bone	13.9 ± 1.3	-11.0 ± 1.2		2.0	77	1.3	48	
		Delphinus delphis	15	Bone	12.5 ± 1.0	-12.4 ± 0.7	-12.7 ± 0.7	0.8	31	0.0	0	Present study

		Monachus monachus	12	Bone	14.5 ± 0.7	-10.4 ± 0.6	-10.7 ± 0.6	2.6	100	2.0	74	
		Phocoena phocoena	12	Bone	13.8 ± 0.6	-11.6 ± 0.7	-12.0 ± 0.7	1.7	65	0.7	26	
		Sousa teuszii	5	Bone	13.0 ± 1.2	-9.6 ± 0.3	-10.0 ± 0.3	1.4	56	2.7	100	
		Stenella frontalis	4	Bone	13.2 ± 1.0	-12.1 ± 0.3	-12.5 ± 0.3	1.3	50	0.3	11	
		Tursiops truncatus	34	Bone	13.7 ± 1.3	-11.0 ± 1.3	-11.4 ± 1.3	2.0	77	1.3	48	
**Western South Atlantic**												
Northern Brazil	*Globicephala macrorhynchus*	*Delphinus sp.*	2	Bone	12.3 ± 0.2	-12.0 ± 0.8		1.3	57	1.2	55	[5]
	Grampus griseus	*Pseudorca crassidens*	1	Bone	13.3	-11.0		2.3	100	2.2	100	
	Peponocephala electra	*Lagenodelphis hosei*	1	Bone	13.3	-13.0		2.3	100	0.2	9	
	Physeter macrocephalus	*Sotalia guianensis*	32	Bone	11.5 ± 1.0	-12.2 ± 0.6		0.5	22	1.0	50	
		Stenella attenuata	2	Bone	11.0 ± 0.4	-13.2 ± 0.1		0.0	0	0.0	0	
		Steno bredanensis	9	Bone	12.2 ± 1.0	-12.0 ± 0.8		1.2	52	1.2	55	
		Tursiops truncatus	6	Bone	13.0 ± 1.3	-11.3 ± 0.7		2.0	96	1.9	86	
Southern Brazil	*Grampus griseus*	*Delphinus sp.*	2	Muscle	12.3 ± 0.4	-15.6 ± 0.6		0.9	23	1.5	58	[6]
		Pseudorca crassidens	2	Muscle	15.3 ± 0.9	-14.5 ± 0.3		3.9	100	2.6	100	
		Lagenodelphis hosei	10	Muscle	12.6 ± 1.0	-16.5 ± 0.3		1.2	31	0.6	23	
		Sotalia guianensis	44	Muscle	14.1 ± 0.6	-14.6 ± 0.9		2.7	69	2.5	96	
		Stenella attenuata	2	Muscle	11.4 ± 0.2	-15.8 ± 0.2		0.0	0	1.3	50	
		Stenella longirostris	1	Muscle	12.1	-17.1		0.7	18	0.0	0	
		Stenella frontalis	13	Muscle	13.5 ± 1.0	-16.0 ± 0.4		2.1	54	1.1	42	
		Steno bredanensis	3	Muscle	14.4 ± 0.3	-14.9 ± 0.5		3.0	77	2.2	85	
		Tursiops truncatus	7	Muscle	14.9 ± 1.7	-15.5 ± 0.5		3.5	90	1.6	62	
Uruguay		*Arctocephalus australis*	33	Bone	19.1 ± 0.9	-14.7 ± 0.5	-15.0 ± 0.6	2.8	58	1.3	39	Present study
		Otaria byronia	29	Bone	20.8 ± 0.8	-12.8 ± 0.9	-13.2 ± 0.9	4.5	94	2.8	84	
		Lagenodelphis hosei	30	Bone	16.3 ± 1.3	-14.8 ± 1.0	-15.4 ± 0.9	0.0	0	0.9	27	
		Delphinus delphis	6	Bone	16.7 ± 1.3	-14.2 ± 0.9	-14.7 ± 0.9	0.4	8	1.6	5	
		Pontoporia blainvillei	25	Bone	21.1 ± 0.6	-14.6 ± 1.0	-15.0 ± 1.0	4.8	100	1.3	39	
		Pseudorca crassidens	14	Bone	18.1 ± 2.0	-13.7 ± 1.8	-14.1 ± 1.7	1.8	38	2.2	67	
		Phocoena spinipinnis	5	Bone	20.2 ± 0.9	-15.9 ± 1.9	-16.3 ± 1.9	3.9	81	0.0	0	
		Tursiops truncatus	5	Bone	18.7 ± 1.4	-12.2 ± 0.8	-13.0 ± 0.5	2.4	50	3.3	100	
Southern Argentina		*Arctocephalus australis*	6	Bone	19.0 ± 0.2	-13.2 ± 0.7		8.9	83	4.7	66	[7]
		Otaria byronia	41	Bone	20.8 ± 1.1	-12.7 ± 0.9		10.7	100	5.2	73	
	Grampus griseus	*Cephalorhynchus commersonii*	121	Bone	17.2 ± 0.7	-12.7 ± 1.3		7.1	66	5.2	73	[8]
		Pseudorca crassidens	27	Bone	13.1 ± 0.7	-12.8 ± 0.4		3.0	28	5.1	72	
		Lagenorhynchus australis	39	Bone	19.3 ± 1.6	-10.8 ± 1.2		9.2	86	7.1	100	
		Lagenorhynchus cruciger	5	Bone	10.2 ± 0.8	-17.2 ± 1.0		0.1	1	0.7	10	
		Phocoena dioptrica	87	Bone	10.1 ± 0.9	-17.9 ± 1.2		0.0	0	0.0	0	
		Phocoena spinipinnis	7	Bone	17.9 ± 0.7	-13.0 ± 1.0		7.8	73	4.9	69	
		Lissodelphis peronii	37	Bone	15.3 ± 2.4	-12.9 ± 0.9		5.2	49	5.0	70	

Table S2. Bayesian standard ellipse areas (SEA_B) and their respective 95% credibility intervals (CI) of the marine mammal species from Uruguay and Mauritania. Isotopic overlap areas between species (calculated with SEA_C) and the respective percentage of overlap surface for each species (estimated from overlap areas) were shown only when > 0 (see isotopic niche areas in Fig. 4). Species: South American sea lion (Of), common dolphin (Dd), bottlenose dolphin (Tt), Burmeister's porpoise (Ps), Fraser's dolphin (Lh), false killer whale (Pc), South American fur seal (Aa), franciscana dolphin (Pb), Mediterranean monk seal (Mm), harbor porpoise (Pp), Atlantic spotted dolphin (Sf) and Atlantic humpback dolphin (St).

Community	Species	SEA_B (‰^{2})	95% CI (‰^{2})	Species	Overlap area (‰^{2})	% Overlap area for species	
**Uruguay**	Of	2.46	1.63-3.39	Of vs. Tt	0.16	6.72	5.27
	Dd	4.10	1.47-7.53	Of vs. Pb	0.05	2.10	2.45
	Tt	3.64	1.17-7.04	Dd vs. Lh	1.93	45.82	51.03
	Ps	5.91	1.91-11.50	Dd vs. Pc	2.59	61.48	22.15
	Lh	3.81	2.55-5.21	Tt vs. Pc	2.37	78.04	20.27
	Pc	10.60	5.69-16.50	Ps vs. Pc	0.82	11.90	7.01
	Aa	1.71	1.17-2.30	Ps vs. Aa	0.47	6.82	30.26
	Pb	2.20	1.42-3.09	Ps vs. Pb	0.76	11.03	37.19
				Lh vs. Pc	1.07	28.29	9.15
				Pc vs. Aa	1.55	13.26	99.80
**Mauritania**	Mm	1.71	0.86-2.75	Mm vs. Tt	0.95	72.48	16.64
	Dd	2.56	1.40-3.89	Mm vs. Pp	0.01	0.76	0.63
	Tt	5.57	3.82-7.46	Mm vs. St	0.01	0.76	0.85
	Pp	1.90	0.94-3.02	Dd vs. Tt	0.28	11.68	4.91
	Sf	2.81	0.78-5.75	Dd vs. Pp	0.09	3.75	5.67
	St	2.68	0.84-5.22	Dd vs. Sf	0.95	39.63	62.26
				Tt vs. Pp	1.56	27.33	98.33
				Tt vs. Sf	0.77	13.49	50.46
				Tt vs. St	0.35	6.13	29.69
				Pp vs. Sf	0.57	35.93	37.36

Table S3. Marine mammal prey species based on stomach and scat content analysis.

Predator	Diet/Foraging habits preference	Prey	Source
Common dolphin (*Delphinus delphis*)	Pelagic, neritic (Clupeids and small gaodids) and oceanic (myctophids) fishes; squid	*Atherina presbyter, Paralepis, Scomberesox saurus, Exocoetidae, Belone belone, Petalichthys capensis,* Engraulidae, *Sprattus, Sardina pilchardus, Engraulis encrasicolus, Etrumeus whiteheadi, Sardinops, Engraulis capensis, Hilsa kelee, Engraulis japonicus, Thryssa vitrirostris, Micromesistius poutassou, Gadiculus argenteus, Gaidropsarus, Merlangius merlangus, Trisopterus, Merluccius merluccius, Merluccius capensis, Bregmaceros, Notoscopelus kroyeri, Ceratoscopelus maderensis, Diaphus, Lampanyctodes hectoris, Myctophum, Symbolophorus boops, Scopelopsis, Raneya brasiliensis,* Osmeridae, *Argentina, Pagrus pagrus, Cynoscion guatucupa, Trachurus lathami, Stellifer, Scomber scombrus, Trachurus capensis, Capros aper, Cepola macrophthalma, Boops boops, Diplodus vulgaris, Hyperoplus lanceolatus, Scomber japonicus, Scomber, Deltentosteus quadrimaculatus, Gobius, Callionymus lyra, Liza, Ambassis kopsii, Pomatomus saltatrix, Pomadasys commersonnii, Pagellus bellottii, Sarpa salpa, Argyrosomus thorpei, Otolithes ruber, Trachurus delagoa, Trichiurus lepturus, Dicologlossa cuneata, Arnoglossus imperialis, Arnoglossus laterna, Microchirus variegatus, Paralichthys olivaceus, Sepia officinalis, Semirossia tenera, Sepiola atlántica, Maurolicus muelleri, Stomias boa, Chauliodus sloani, Macroramphosus, Illex coindetii, Loligo opalescens, Loligo plei, Loligo sanpaulensis, Thysanoteuthis rhombus, Loligo vulgaris, Octopoteuthis sicula, Brachioteuthis, Chiroteuthis, Lycoteuthis lorigera, Ommastrephes bartramii.*	9-17
Atlantic white-sided dolphin (*Lagenorhynchus acutus*)	Neritic fishes (no ocean fishes)	Salmonidae, *Clupea harengus, Scomber scombrus, Gadiculus argenteus, Micromesistius poutassou, Ammodytes americanus, Osmerus mordax, Merluccius bilinearis, Illex illecebrosus, Gadus.*	13,18,19
White-beaked dolphin (*Lagenorhynchus albirostris*)	Gadoids, benthic fishes (no ocean fishes)	*Gadus morhua, Melanogrammus aeglefinus, Trisopterus minutus, Trisopterus luscus, Merlangius merlangus, Mallotus villosus, Merluccius merluccius, Eleginus, Clupea harengus, Trisopterus esmarkii, Phycis blennoides, Raniceps raninus, Eutrigla gurnardus, Trachurus trachurus, Scomber scombrus, Hippoglossoides platessoides, Solea solea, Eledone cirrhosa, Pollachius pollachius, Sprattus sprattus, Ammodytes marinus, Ammodytes tobianus, Hyperoplus lanceolatus, Pomatoschistus microps, Pomatoschistus minutus, Pomatoschistus norvegicus, Pomatoschistus pictus, Buglossidium luteum, Limanda limanda, Pleuronectes platessa, Callionymus lyra, Enchelyopus cimbrius, Mullus surmuletus, Osmerus eperlanus, Trachinus draco,* Sepiolidae.	13,20,21
Pantropical spotted dolphin (*Stenella attenuata*)	Oceanic pelagic fishes	Congridae, Notosudidae, Scopelarchidae, Chlorophthalmidae, *Lestidiops similis, Lestrolepis intermedia, Paralepididae, Exocoetus monocirrhus, Exocoetus volitans, Hemirhamphidae, Tylosurus acus melanotus, Engraulis japonicus, Bregmaceros bathymaster, Bregmaceros nectabanus, Benthosema panamense, Ceratoscopelus warmingii, Diaphus mollis, Diaphus splendidus, Hygophum proximum, Hygophum reinhardtii, Lampadena luminosa, Lampanyctus festivus, Lampanyctus omostigma, Lampanyctus parvicauda, Myctophum asperum, Myctophum nitidulum, Myctophum spinosum, Nannobrachium idostigma, Notoscopelus resplendens, Parvilux ingens, Tarletonbeania crenularis, Benthosema fibulatum, Diaphus jenseni, Diaphus schmidti, Diaphus watasei, Lampanyctodes hectoris, Lampanyctus australis, Myctophum obtusirostre, Symbolophorus evermanni, Diaphus garmani,* Alloposidae, *Tremoctopus violaceus,* Bathylagidae, Microstomatidae, Opisthoproctidae, *Glossanodon semifasciatus, Xenodermichthys, Cubiceps baxteri, Cubiceps paradoxus, Cubiceps pauciradiatus, Synagrops japonicus, Malakichthys elegans, Priacanthus macracanthus, Apogon carinatus, Decapterus macrosoma, Decapterus maruadsi, Decapterus russelli, Rexea prometheoides, Trichiurus lepturus, Auxis thazard, Scomber australasicus, Scomber japonicus, Pampus argenteus, Decapterus macarellus,* Scorpaenidae, Melamphaidae, *Vinciguerria lucetia, Sigmops elongatus, Diplophos taenia, Polyipnus indicus, Bonapartia pedaliota, Lagocephalus, Thysanoteuthis rhombus, Architeuthidae, Chiroteuthis, Loligo vulgaris, Lycoteuthis, Ommastrephes bartramii, Todarodes angolensis, Todaropsis eblanae, Selenoteuthis, Enoploteuthis chunii, Abraliopsis lineata, Onychoteuthis banksia, Histioteuthis miranda, Chtenopteryx, Sthenoteuthis oualaniensis, Eucleoteuthis, Ornithoteuthis volatilis, Mastigoteuthis, Galiteuthis pacifica, Galiteuthis armata, Histioteuthis hoylei, Octopoteuthis deletron, Pholidoteuthis boschmai.*	14,22,23

Striped dolphin (*Stenella coeruleoalba*)	Pelagic fishes (generally oceanic but also neritic)	*Brachioteuthis riisei, Todarodes sagittatus, Abraliopsis pfefferi, Onychoteuthis banksia, Gadiculus argenteus, Atherina presbyter, Abraliopsis pfefferi, Ancistrocheirus lesueurii, Abralia veranyi, Todaropsis eblanae, Octopoteuthis sicula, Chiroteuthis veranyi, Notoscopelus kroyeri, Ceratoscopelus maderensis, Scomberesox saurus, Stomias boa, Chauliodus sloani, Xenodermichthys copei, Loligo plei, Maurolicus muelleri, Argyropelecus olfersii, Bathylagus greyae, Benthosema glaciale, Lobianchia gemellarii, Myctophum punctatum, Symbolophorus veranyi, Electrona risso, Macroparalepis affinis, Arctozenus risso, Paralepis brevirostris, Paralepis coregonoides, Serrivomer beanie, Nesiarchus nasutus, Cubiceps gracilis, Ancistroteuthis lichtensteini, Gonatus steenstrupi, Pholidoteuthis, Histioteuthis bonnellii, Histioteuthis reversa, Histioteuthis corona, Chirothauma, Teuthowenia megalops, Heteroteuthis dispar, Eledone, Rossia, Sepiola atlantica, Gaidropsarus, Melanogrammus aeglefinus*, Gadidae, Gobiidae, Scombridae, *Ammodytidae, Decapterus, Sprattus, Trachurus capensis, Merluccius merluccius, Loligo vulgaris, Octopus, Sthenoteuthis*, Argentinidae, Gonostomatidae, Mugilidae, Nemipteridae, Pleuronectidae, Priacanthidae, Zeidae, Lycoteuthidae, Sepiidae, *Micromesistius poutassou, Belone belone, Boops, Engraulis encrasicolus, Hygophum, Diaphus rafinesquii, Lampanyctus crocodilus, Sepietta oweniana, Neorossia caroli, Loligo forbesii, Loligo media, Illex coindetii, Scaeurgus unicirrhus.*	10,13,14,16,17,24-28
Atlantic spotted dolphin (*Stenella frontalis*)	Neritic and benthic fishes; few oceanic fishes	*Porichthys porosissimus, Engraulis anchoíta, Pogonias cromis, Chloroscombrus chrysurus, Cynoscion guatucupa, Cynoscion jamaicensis, Micropogonias furnieri, Loligo plei, Clupeidae*, Hemirhamphidae, Holocentridae, Exocoetidae, *Syacium, Trachurus lathami, Trichiurus lepturus, Mugil, Argonauta, Loligo sanpaulensis, Thysanoteuthis rhombus, Octopus vulgaris, Ornithoteuthis antillarum, Loligo plei.*	16,29
Bottlenose dolphin (*Tursiops truncatus*)	Pelagic and demersal fishes (in equal parts), few ocean fishes	*Gadus morhua, Hippoglossoides platessoides, Limanda limanda, Melanogrammus aeglefinus, Merlangius merlangus, Merluccius merluccius, Molva molva, Myoxocephalus scorpius, Pleuronectes platessa, Pollachius virens, Porichthys porosissimus, Sprattus sprattus, Loligo plei, Octopus vulgaris, Bairdiella chrysoura, Cynoscion regalis, Micropogonias undulatus, Leiostomus xanthurus, Trachurus delagoa, Pomadasys olivaceus, Pagellus bellottii, Scomber japonicus, Sepia officinalis, Sardinops sagax, Engraulis ringens, Caranx hippos, Lagodon rhomboides, Elops saurus, Orthopristis chrysoptera, Mugil cephalus, Opsanus beta, Archosargus probatocephalus, Ophichthus gomesii, Gerres cinereus, Menticirrhus, Pogonias cromis, Sciaenops odatus, Stellifer lanceolatus, Anchoa hepsetus, Brevoortia tyrannus, Diplodus bolbrook, Paralichthys, Citharichthys, Urophycis, Synodus foetens, Morone saxatilis, Pomatomus saltatrix, Trinectes maculatus, Todarodes sagittatus, Illex coindetii, Eledone cirrhosa, Congridae, Ariidae, Exocoetidae, Priacanthidae, Bothidae, Syacium, Trichiurus lepturus, Loligo sanpaulensis, Loligo plei, Trachurus capensis, Liza richardsonii, Lepidopus caudatus, Loligo vulgaris, Lycoteuthis, Todaropsis eblanae.*	11,13,14,16,29-32
South American sea lion (*Otaria byronia*)	Demersal fishes and some neritic fishes	*Merluccius hubbsi, Raneya brasiliensis, Engraulis anchoita, Patagonotothen cornucola, Paralichthys isosceles, Triathalassothia argentina, Genypterus blacodes, Stromateus brasiliensis, Enteroctopus megalocyathus, Illex argentinus, Loligo gahi, Octopus tehuelchus, Loligo sanpaulensis, Pleoticus muelleri, Munida subrugosa, Cynoscion guatucupa, Engraulis anchoita, Anchoa marinii, Trichiurus lepturus, Urophycis brasiliensis, Micropogonias furnieri, Conger japonicus*, Cephalopods.	33,34
South American fur seal (*Arctocephalus australis*)	Demersal fishes and some neritic fishes	*Cynoscion guatucupa, Engraulis anchoita, Anchoa marinii, Trichiurus lepturus, Micropogonias furnieri, Trachurus lathami, Merluccius hubbsi, Cynoscion guatucupa, Engraulis anchoita, Menticirrhus americanus, Umbrina canosai, Urophycis brasiliensis, Raneya brasiliensis, Patagonotothen spp.*, Cephalopods, *Illex argentinus, Loligo gahi, Pleoticus muelleri, Munida gregaria.*	35,36
Gray seal (*Halichoerus grypus*)	Demersal fishes and some neritic fishes	*Clupea harengus, Sprattus sprattus, Coregonus lavaretus, Ammodytes spp., Zoarces viviparus, Gadus morhua*, Cyprinidae, *Salmo spp.*, Pleuronectiforme, *Perca fluviatilis, Anguilla anguilla, Osmerus eperlanus*, Cottidae, Gobiidae, *Lota lota, Esox lucius.*	37,38

References

1. Das, K. *et al.* Marine mammals from northeast atlantic: relationship between their trophic status as determined by d^{13}C and d^{15}N measurements and their trace metal concentration. *Mar. Environ. Res.* **56**, 349-365 (2003).
2. Das, K., Lepoint, G., Leroy, Y. & Bouquegneau, J. M. Marine mammals from the southern North Sea: feeding ecology data from d^{13}C and d^{15}N measurements. *Mar. Ecol. Prog. Ser.* **263**, 287-298 (2003).
3. Mèndez-Fernandez, P. *et al.* Foraging ecology of five toothed whale species in the Northwest Iberian Peninsula, inferred using carbon and nitrogen isotope ratios. *J. Exp. Mar. Biol. Ecol.* **413**, 150–158 (2012).
4. Pinela, A. M., Borrell, A., Cardona, L. & Aguilar, A. Stable isotope analysis reveals habitat partitioning among marine mammals off the NW African coast and unique trophic niches for two globally threatened species. *Mar. Ecol. Prog. Ser.* **416**, 295–306 (2010).
5. Costa, A. F., Botta, S., Siciliano, S. & Giarrizzo, T. Resource partitioning among stranded aquatic mammals from Amazon and northeastern coast of Brazil revealed through carbon and nitrogen stable isotopes. *Sci. Rep.* **10**, 12897 (2020).
6. Bisi, T. L. *et al.* Trophic relationships and habitat preferences of delphinids from the southeastern Brazilian coast determined by carbon and nitrogen stable isotope composition. *PLoS One* **8**, e82205 (2013).
7. Saporiti, F. *et al.* Resource partitioning among air-breathing marine predators: are body size and mouth diameter the major determinants? *Mar. Ecol.* **37**, 957-969 (2016).

8. Ricciardelli, L., Newsome, S. D., Fogel, M. L. & Goodall, R. N. Isotopic assessment of prey and habitat preferences of a cetacean community in the southwestern South Atlantic Ocean. *Mar. Ecol. Prog. Ser.* **418**, 235-248 (2010).

9. Meynier, L. *et al.* Intraspecific dietary variation in the short-beaked common dolphin *Delphinus delphis* in the Bay of Biscay: importance of fat fish. *Mar. Ecol. Prog. Ser.* **354**, 277-287 (2008).

10. Hassani, S., Antoine, L. & Ridoux, V. Diets of albacore, *Thunnus alalunga*, and dolphins, *Delphinus delphis* and *Stenella coerulaeoalba*, caught in the northeast Atlantic albacore drift-net fishery: a progress report. *J. Northwest Atl. Fish. Sci.* **22**, 119-123 (1997).

11. Heithaus, M. R. Predator–prey and competitive interactions between sharks (order Selachii) and dolphins (suborder Odontoceti): a review. *J. Zool.* **253**, 53-68 (2001).

12. Young, D. D. & Cockcroft, V. Diet of common dolphins (*Delphinus delphis*) off the south-east coast of southern Africa: opportunism or specialization? *J. Zool.* **234**, 41-53 (1994).

13. Culik, B. M. Review of small cetaceans: distribution, behaviour, migration and threats. 84 (United Nations Environment Program (UNEP), Convention on Migratory Species (CMS), Bonn, Germany, 2004).

14. Sekiguchi, K., Klages, N. & Best, P. Comparative analysis of the diets of smaller odontocete cetaceans along the coast of southern Africa. *S. Afr. J. Mar. Sci.* **12**, 843-861 (1992).

15. Silva, M. Diet of common dolphins, *Delphinus delphis*, off the Portuguese continental coast. *J. Mar. Biol. Assoc. U. K.* **79**, 531-540 (1999).

16. Melo, C. *et al.* Feeding habits of delphinids (Mammalia: Cetacea) from Rio de Janeiro state, Brazil. *J. Mar. Biol. Assoc. U. K.* **90**, 1509-1515 (2010).

17. Perrin, W. F., Wursig, B. & Thewissen, J. G. M. *Encyclopedia of marine mammals*. (Academic Press, 2009).
18. Gaskin, D. E. *Ecology of whales and dolphins*. (Heinemann Educational Books, 1982).
19. Jefferson, T. A., Webber, M. A. & Pitman, R. L. *Marine mammals of the world: A comprehensive guide to their identification*. (Academic Press, 2015).
20. Canning, S. J. *et al.* Seasonal distribution of white-beaked dolphins (*Lagenorhynchus albirostris*) in UK waters with new information on diet and habitat use. *J. Mar. Biol. Assoc. U. K.* **88**, 1159-1166 (2008).
21. Jansen, O. E., Leopold, M. F., Meesters, E. H. & Smeenk, C. Are white-beaked dolphins *Lagenorhynchus albirostris* food specialists? Their diet in the southern North Sea. *J. Mar. Biol. Assoc. U. K.* **90**, 1501-1508 (2010).
22. Wang, M. C., Walker, W. A., Shao, K. T. & Chou, L.-S. Feeding habits of the pantropical spotted dolphin, *Stenella attenuata*, off the eastern coast of Taiwan. *Zool. Stud.* **42**, 368-378 (2003).
23. Wang, M. C., Shao, K. T., Huang, S. L. & Chou, L. S. Food partitioning among three sympatric odontocetes (*Grampus griseus*, *Lagenodelphis hosei*, and *Stenella attenuata*). *Mar. Mamm. Sci.* **28**, E143-E157 (2012).
24. Blanco, C., Aznar, J. & Raga, J. A. Cephalopods in the diet of the striped dolphin *Stenella coeruleoalba* from the western Mediterranean during an epizootic in 1990. *J. Zool.* **273**, 151-158 (1995).
25. Ringelstein, J. *et al.* Food and feeding ecology of the striped dolphin, *Stenella coeruleoalba*, in the oceanic waters of the north-east Atlantic. *J. Mar. Biol. Assoc. U. K.* **86**, 909-918 (2006).

26. Santos, M. *et al.* Strandings of striped dolphin *Stenella coeruleoalba* in Scottish waters (1992–2003) with notes on the diet of this species. *J. Mar. Biol. Assoc. U. K.* **88**, 1175-1183 (2008).

27. Spitz, J., Richard, E., Meynier, L., Pusineri, C. & Ridoux, V. Dietary plasticity of the oceanic striped dolphin, *Stenella coeruleoalba*, in the neritic waters of the Bay of Biscay. *J. Sea Res.* **55**, 309-320 (2006).

28. Würtz, M. & Marrale, D. Food of striped dolphin, *Stenella coeruleoalba*, in the Ligurian Sea. *J. Mar. Biol. Assoc. U. K.* **73**, 571-578 (1993).

29. dos Santos, R. A. & Haimovici, M. Cephalopods in the diet of marine mammals stranded or incidentally caught along southeastern and southern Brazil (21–34 S). *Fish. Res.* **52**, 99-112 (2001).

30. Barros, N. B. & Wells, R. S. Prey and feeding patterns of resident bottlenose dolphins (*Tursiops truncatus*) in Sarasota Bay, Florida. *J. Mammal.* **79**, 1045-1059 (1998).

31. Gannon, D. P. & Waples, D. M. Diets of coastal bottlenose dolphins from the US mid-Atlantic coast differ by habitat. *Mar. Mamm. Sci.* **20**, 527-545 (2004).

32. González, A., López, A., Guerra, A. & A., B. Diets of marine mammals stranded on the northwestern Spanish Atlantic coast with special reference to Cephalopoda. *Fish. Res.* **21**, 179-191 (1994).

33. Koen Alonso, M., Crespo, E. A., Pedraza, S. N., Garcia, N. A. & Coscarella, M. A. Food habits of the South American sea lion, *Otaria flavescens,* off Patagonia, Argentina. *Fish. Bull.* **98**, 250–263 (2000).

34. Szteren, D., Naya, D. E. & Arim, M. Overlap between pinniped summer diet and artisanal fishery catches in Uruguay. *Lat. Am. J. Aquat. Mamm.* **3**, 119–125 (2004).

35. Naya, D. E., Arim, M. & Vargas, R. Diet of South American fur seals (*Arctocephalus australis*) in Isla de Lobos, Uruguay. *Mar. Mamm. Sci.* **18**, 734–745 (2002).

36. Crespo, E. A., García, N. A., Dans, S. & Pedraza, S. N. in *Atlas de sensibilidad ambiental de la costa y el mar Argentino* (ed D. Boltovskoy) 1–9 (Secretaría de Ambiente y Desarrollo Sustentable de la Nación, 2008).

37. Lundström, K., Hjerne, O., Lunneryd, S.-G. & Karlsson, O. Understanding the diet composition of marine mammals: grey seals (*Halichoerus grypus*) in the Baltic Sea. *ICES J. Mar. Sci.* **67**, 1230–1239 (2010).

38. Lundström, K., Hjerne, O., Alexandersson, K. & Karlsson, O. Estimation of grey seal (Halichoerus grypus) diet composition in the Baltic Sea. *NAMMCO Sci. Publ.* **6**, 177-196 (2007).
